# Head and Neck Schwannomas: A Surgical Challenge—A Series of 5 Cases

**DOI:** 10.1155/2018/4074905

**Published:** 2018-03-04

**Authors:** Ishtyaque Ansari, Ashfaque Ansari, Arjun Antony Graison, Anuradha J. Patil, Hitendra Joshi

**Affiliations:** ^1^Department of Neurosurgery, MGM Medical College & Hospital, Aurangabad, India; ^2^Department of ENT, MGM Medical College & Hospital, Aurangabad, India; ^3^Department of Plastic Surgery, MGM Medical College & Hospital, Aurangabad, India

## Abstract

**Background:**

Schwannomas, also known as neurilemmomas, are benign peripheral nerve sheath tumors. They originate from any nerve covered with schwann cell sheath. Schwannomas constitute 25–45% of tumors of the head and neck. About 4% of head and neck schwannomas present as a sinonasal schwannoma. Brachial plexus schwannoma constitute only about 5% of schwannomas. Cervical vagal schwannomas constitute about 2–5% of neurogenic tumors.

**Methods:**

We present a case series of 5 patients of schwannomas, one arising from the maxillary branch of trigeminal nerve in the maxillary sinus, second arising from the brachial plexus, third arising from the cervical vagus, and two arising from cervical spinal nerves.

**Result:**

Complete extracapsular excision of the tumors was achieved by microneurosurgical technique with preservation of nerve of origin in all except one.

**Conclusion:**

Head and neck schwannoma though rare should be considered as a differential diagnosis of a unilateral slow growing mass in the head and neck region, particularly in an adult. Schwannomas are always a diagnostic dilemma as they are asymptomatic for long time, and histopathology is the gold standard for diagnosis. As a rule, treatment is surgical and dictated by the location of the tumor and nerve of origin. Due to its rarity, complex anatomical location and morbidity risk postexcision, they can pose a formidable challenge to surgeons. This study aims to describe the presentation, workup, surgical technique, and outcome.

## 1. Introduction

Neurilemmoma also known as schwannoma is a benign tumor of nerve sheath origin, arising from any nerve covered with a schwann cell sheath, which includes the cranial nerves (except for optic and olfactory), the spinal nerves, and autonomic nervous system [1, 2].

Schwannomas occurring in the head and neck region are frequent, with 25–45% of all reported schwannomas being found in this region [1].

We present our surgical experience with five such tumors, one arising from the maxillary branch of the trigeminal nerve in the maxillary sinus, second arising from the brachial plexus, third arising from the cervical vagus, and two arising from cervical spinal nerves. The clinical presentation, surgical treatment, and outcomes of patients with this pathology are described.

## 2. Materials and Methods

A retrospective analysis was done at MGM Medical College and Hospital, Aurangabad, from January 2015 to December 2016. Of the five patients, two were female and three were males, with a mean age of 33 years (ranging from 23 to 40 years). Complete excision of the tumors was achieved by extracapsular excision using microneurosurgical technique with operating microscope. Preservation of nerve of origin was achieved in all except one where conventional extracapsular excision technique was applied. Surgical observation, histopathology, and immunochemistry confirmed the tumors to be benign schwannomas in all five cases. There was no recurrence in any of the five patients (on clinical examination) at one year follow-up.

### 2.1. Case 1

We describe a rare case of schwannoma arising primarily from the maxillary sinus extending into the orbit and intracranially. A 40-year-old female presented with headache since 2 years, left nostril discharge since 1 year, and left eye progressive vision loss since 1 year.

Diagnostic nasal endoscopy (DNE) revealed left-sided nasal mass, pale, firm, pinkish in colour, not bleeding on touch. Clinical examination revealed reduced vision in the left eye with finger counting less than five feet, with mild proptosis of the left eye. There was no motor or sensory loss with no other cranial nerve dysfunction.

**Figure 1:**
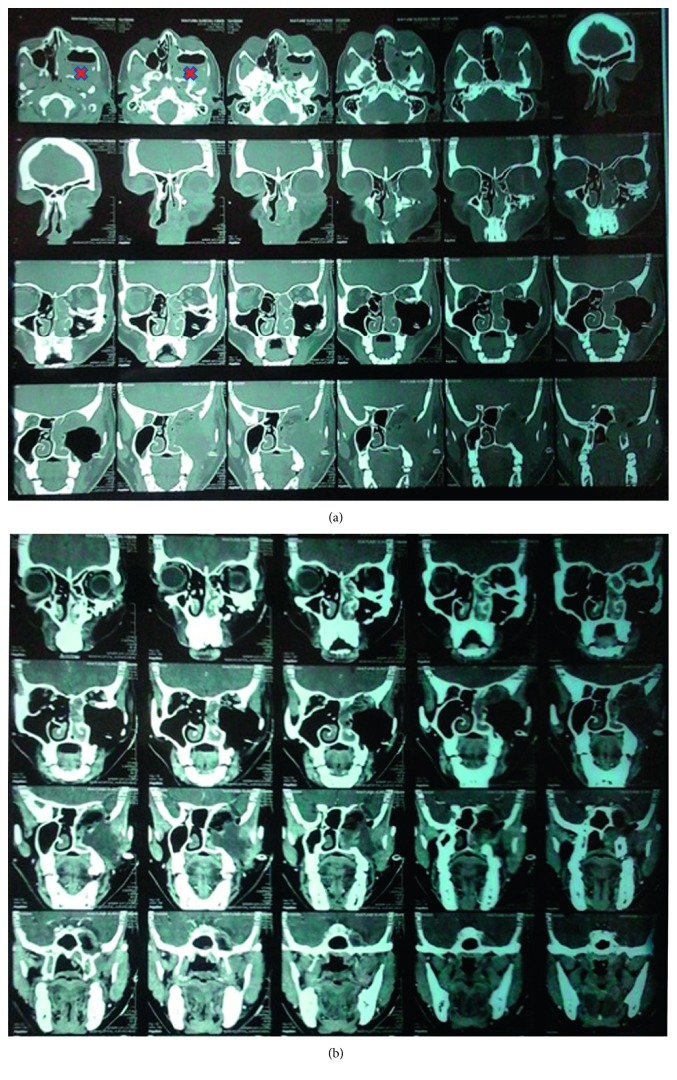
(a) Preoperative CT showing the tumor (^∗^) present in the posterior part of the left maxillary sinus and the left nostril. (b) Postoperative CT.

Contrast-enhanced CT (CECT) scan with cisternography revealed a large lobulated mass of size 81 × 53 × 82 mm in the left zygomatic and masseteric region with intracranial extension (Figure 1(a)). The lesion was causing thinning and pressure erosion of the posterior wall of maxillary sinus, pterygoid plate, posterior wall of orbit, medial wall of sphenoid on the left side, left lesser wing of sphenoid, and was causing thinning of left anterior clinoid process.

Superiorly, mass was extending to middle cranial fossa and displacing left middle temporal lobe. Medially, it was extending to left nasal cavity causing complete blockage. Posteriorly, it was extending to the posterior nasopharyngeal region. Laterally, it was involving and abutting the masseter and pterygoid muscles and intraorbital muscles. Superiorly, it was extending to the infraorbital region and displacing and compressing the left optic nerve. Lamina papyracea and cribriform plate were intact.

Clinical features and CECT were suggestive of malignant tumor. This divisive situation steered us to keep a possibility of a malignant component in the tumor.

**Figure 2:**
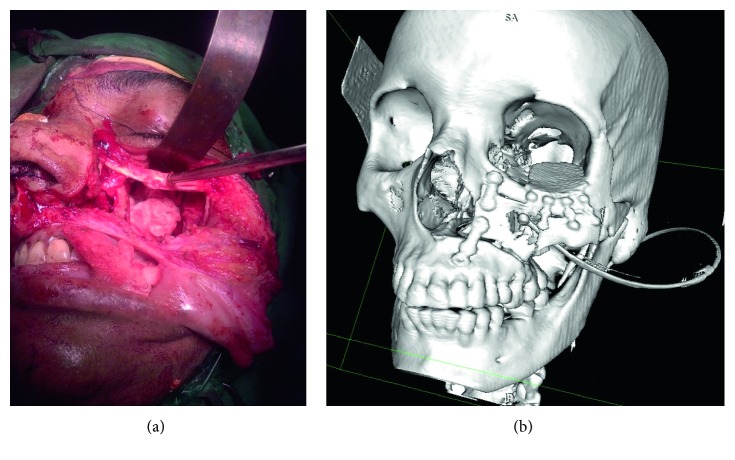
(a) Intraoperative picture. (b) Postoperative 3DCT reconstruction: orbital floor and maxilla reconstruction done with titanium mesh and plate.

The tumor was approached through Weber Furguson incision with subciliary extension. Left suprastructure maxillotomy was done. Tumor was firm, necrotic, moderately vascular, and grayish white (Figure 2(a)) and found arising from the maxillary nerve. Tumour was found to be arising from the left maxilla, breaching it's posterior wall and floor of orbit, extending into the retroorbital portion of orbit through the superior orbital fissure into the left temporal lobe. Total excision of the tumor was carried out. Left temporal bone base was reached, and temporal lobe was identified. Total excision of intracranial portion of tumor was achieved. Bleeding from the left cavernous sinus was controlled with Abgel® and Surgicel®. Orbital floor reconstruction was done with titanium mesh. Maxilla reconstruction was done with titanium mesh and plate (Figure 2(b)). Postoperatively, patient had an uneventful recovery, with minimal left eye vision improvement (Figure 1(b)).

### 2.2. Case 2

**Figure 3:**
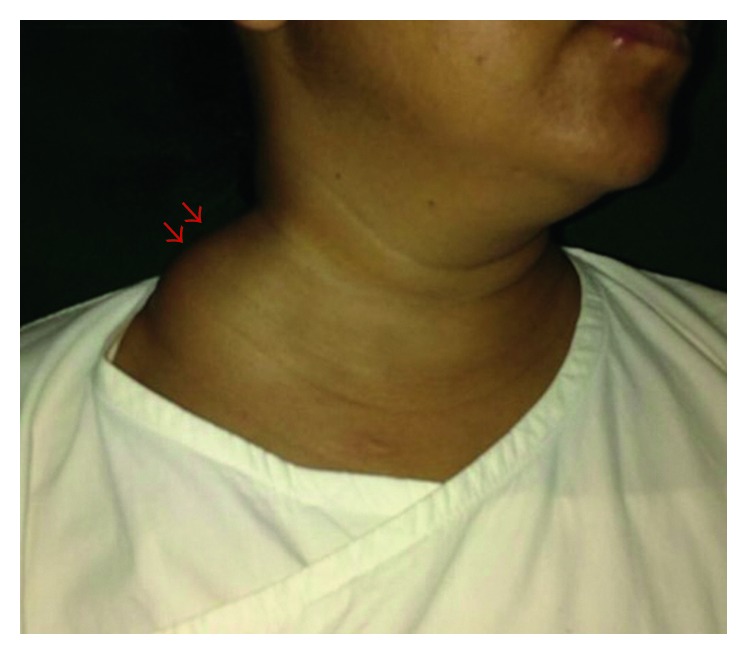
Pre-op image (arrows indicating tumor).

We describe a 35-year-old female who presented with right-sided supraclavicular swelling since 3 years (Figure 3) and complained of paraesthesia over the right upper limb. There was no history of trauma, fever, and systemic illness. The patient had evidence of right C5, C6 radicular pain, and hypoesthesia. The patient had no complains of weakness, loss of function of upper limb. On local examination, a single spherical swelling was present in the right supraclavicular area measuring 5 × 5 cms. Muscle power in all muscles was 5/5, sensations were intact, and there were no signs of wasting. Tinel sign was positive with the patient, and she reported tingling sensation “pins and needles” along the shoulder tip. Spurling's test was negative in this patient.

**Figure 4:**
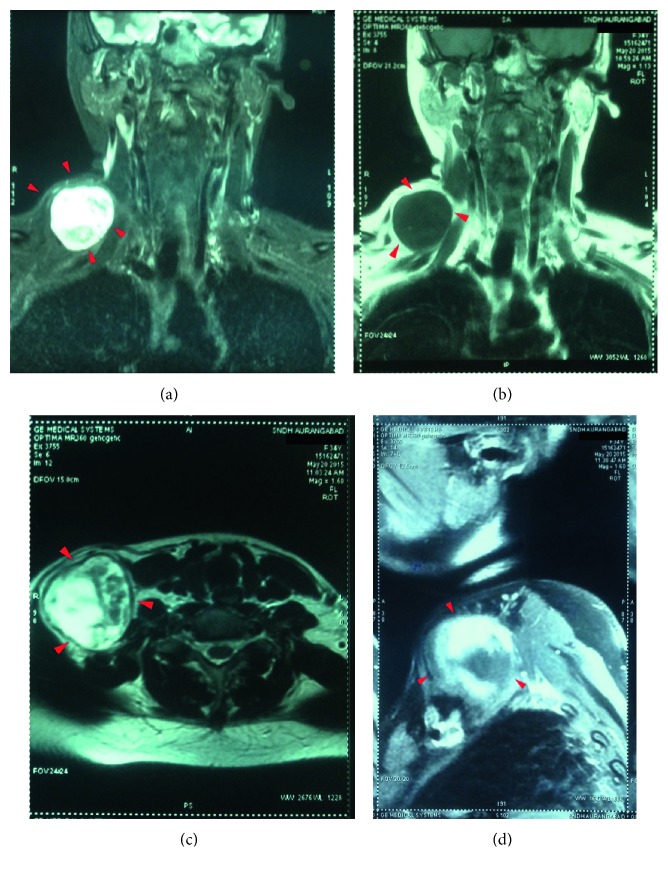
(a) T2-weighted coronal cut. (b) T1-weighted coronal cut. (c) T2-weighted axial cut. (d) Postcontrast T1 sagittal cut.

Pre- and postcontrast MRI neck screening revealed a well-defined, rounded space occupying lesion in the right side of the neck, that was found to be lateral to the neck vessels and superficial to the subclavian vessels. The lesion was hyperintense on T2-weighted images and STIR (short T1 inversion recovery) images and was found to be isointense on T1-weighted images with heterogeneous contrast enhancement in the lesion (Figure 4). These features were suggestive of a neurogenic tumor.

**Figure 5:**
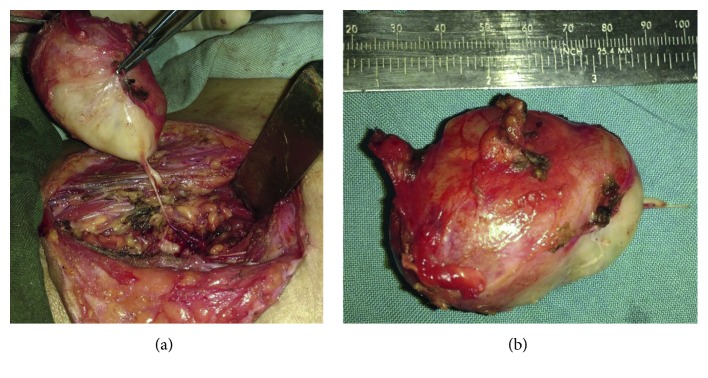
(a) Tumor with nerve attachment. (b) Enucleated tumor with intact capsule.

Under general anaesthesia, the tumor was approached using a V-shaped incision. The brachial plexus was explored. The tumor was found arising from the upper trunk of the brachial plexus and was found to be adherent to the middle and lower trunk of the brachial plexus (Figure 5(a)). The tumor appeared to be greyish white, firm, and globular (Figure 5(b)). The tumor was carefully excised in toto after debulking by microneurosurgical technique. Intra-op nerve stimulator was used for localising the nerve fascicles around the tumor and during excision. After excision, the nerve fascicles were found to be intact. Postoperatively, the patient complained of paraesthesia of the right upper limb which recovered in two weeks.

### 2.3. Case 3

We describe a 23-year-old male patient who presented with right-sided painless neck swelling since one month. There was no history of trauma, fever, and systemic illness. The patient had no complains of weakness, dysphagia, and hoarseness of voice. On local examination, a single ovoid swelling was present in the right carotid triangle, anterior to the sternocleidomastoid, measuring 3 × 2 cms. Swelling did not move on deglutition or tongue protrusion. There was no loss of muscle power in all muscles, no signs of wasting, or neurological deficits.

USG neck showed a hypoechoic lesion seen at bifurcation of the carotid artery. Moderate central and peripheral vascularity was seen within the lesion. Findings were suggestive of a carotid body tumor/nerve sheath tumor.

**Figure 6:**
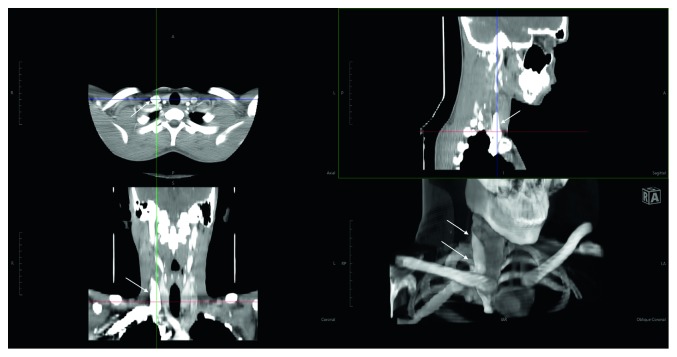
Neck mass (arrows).

CECT neck revealed a well-defined, well-encapsulated, peripherally enhancing soft tissue mass lesion of size 3.4 × 1.8 cm (Figure 6). The mass lesion was noted in the right carotid sheath between the right common carotid artery and internal jugular vein, posterolateral to the right lobe of thyroid. The lesion was seen below the carotid bifurcation. Features were suggestive of a vagal schwannoma.

**Figure 7:**
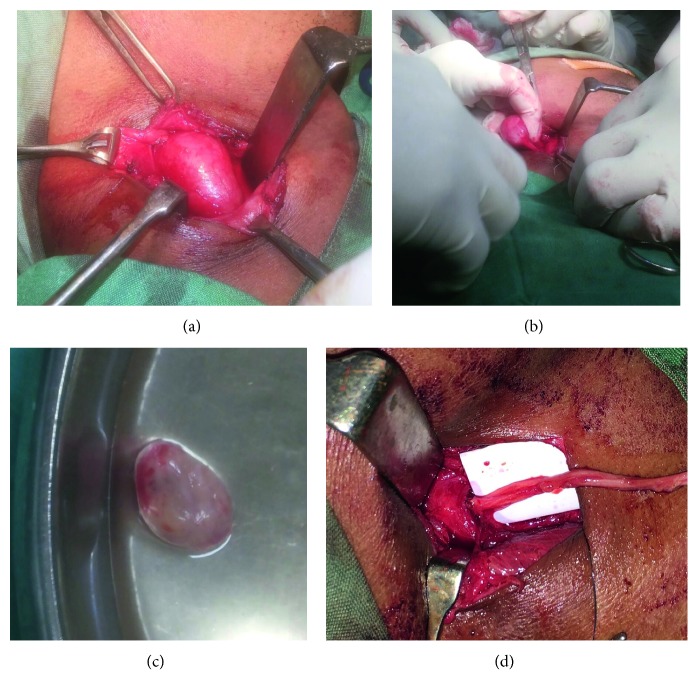
(a) Intra-op tumor excision. (b) Showing the tumor nerve attachment. (c) Enucleated tumor with intact innermost layer of capsule. (d) Vagus reanastomosis using sural nerve graft.

The tumor was approached by an anterior approach along the medial border of sternocleidomastoid. Platysma and fascia were dissected to reach the tumor. There was a medium-sized globular smooth mass, well-encapsulated seen engulfing the vagus nerve (Figures 7(a)–7(c)). Extracapsular tumor excision was done in toto by conventional technique. Vagus was severed in the process. Vagus was reanastomosed, using the sural nerve graft (Figure 7(d)).

Postoperatively, the patient had mild dysphagia which resolved in two days. The patient reported a minimal change in voice. There were no signs of aspiration. Postoperative videolaryngoscopic assessment revealed right vocal cord palsy. The patient's voice improved with speech therapy.

### 2.4. Case 4

We present a 30-year-old male patient who presented with left-sided painless neck swelling since two years. It was insidious in onset and gradually progressed in size. The patient had no complains of weakness, dysphagia, and hoarseness of voice. On local examination, a single ovoid swelling was present in the left carotid triangle, anterior to the sternocleidomastoid, measuring 5 × 3 cms. Swelling did not move on deglutition or tongue protrusion. There was no loss of muscle power in all muscles, no signs of wasting, or neurological deficits.

**Figure 8:**
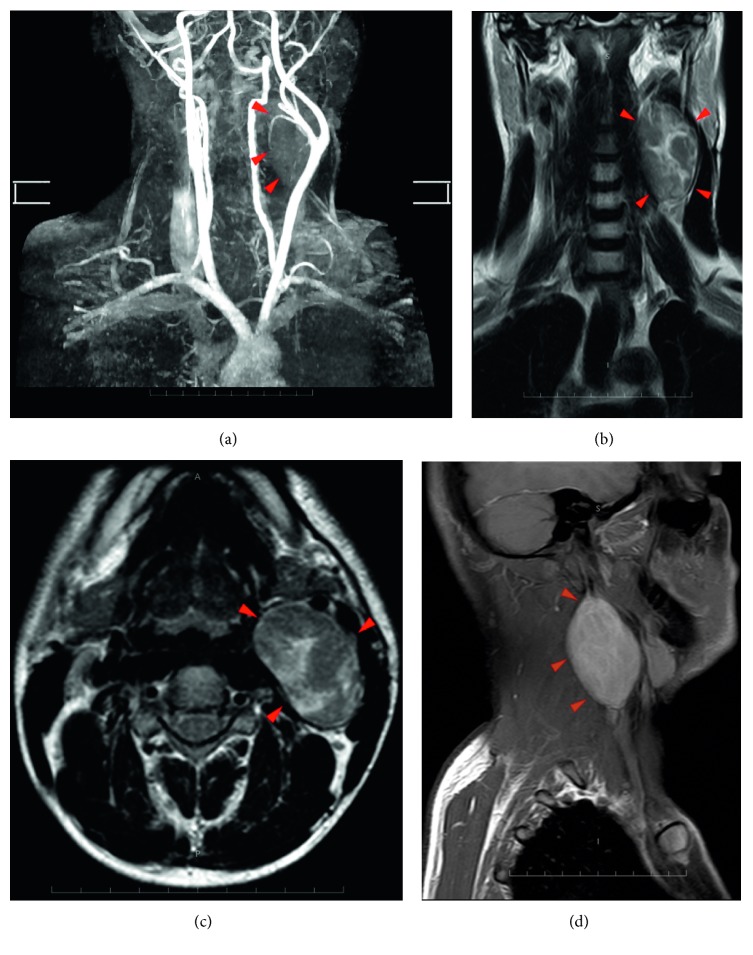
(a) MRI angiography showing splaying of the carotids by the tumor. (b) T2-weighted coronal cut. (c) T2-weighted axial cut. (d) Postcontrast T1 sagittal cut.

MRI neck with angiography and CECT neck revealed a moderately enhancing lesion of size 63 × 45 × 34 mm in left carotid space causing splaying of internal and external carotid artery, anteriorly abutting the submandibular gland with no evidence of encasement of the vessels (Figure 8).

**Figure 9:**
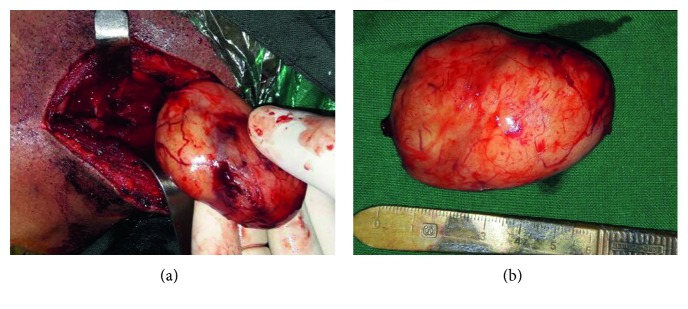
(a) Intraoperative tumor excision. (b) Enucleated tumor with intact innermost layer of capsule.

The tumor was approached by an lateral approach along the lateral border of sternocleidomastoid, 3.5 cms below the auricle. External jugular vein ligated. Tumor was found attached to the C1 spinal nerve. Extracapsular tumor excision was done in toto by microneurosurgical technique (Figure 9). Minivac® drain was placed.

Postoperatively, the patient had mild ptosis of the left eye lid with marginal mandibular nerve paresis and features suggestive of first bite syndrome, all of which improved with physiotherapy. There was no vocal cord paralysis. The patient is currently free of all symptoms at one year follow-up.

### 2.5. Case 5

We present a 35-year-old male patient who presented with right-sided neck swelling since six months. The patient had complaints of right-sided mild ear pain and no complains of weakness, dysphagia, and hoarseness of voice. On local examination, a single ovoid swelling was present in the left carotid triangle, anterior to the sternocleidomastoid, measuring 4 × 4 cms. There was no loss of muscle power in all muscles, no signs of wasting, or neurological deficits.

**Figure 10:**
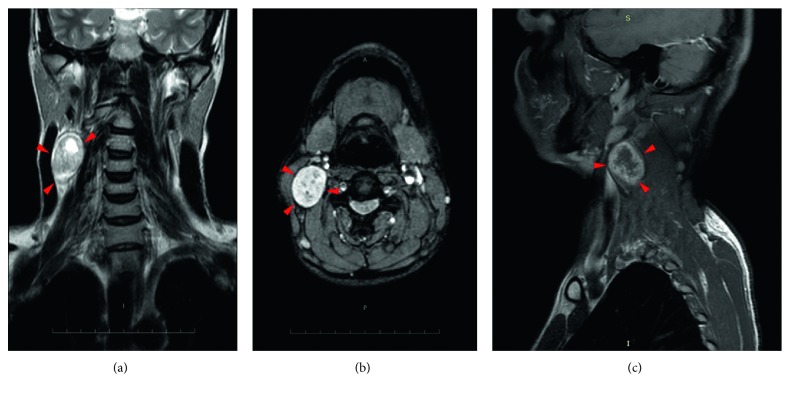
(a) T2-weighted coronal cut. (b) T2-weighted axial cut. (c) Postcontrast T1-weighted sagittal cut.

MRI neck revealed an oval solid lesion of size 42 × 41 × 28 mm in the right carotid triangle, laterally abutting sternocleidomastoid and posteriorly the paraspinal muscle. The lesion was hyperintense on T2W and FLAIR (Fluid Attenuated Inversion Recovery) images and hypointense on T1W images (Figure 10).

The tumor was approached by anterior approach along the medial border of sternocleidomastoid. The tumor was seen involving the cervical spinal nerve. Extracapsular tumor excision was done in toto by microneurosurgical technique, preserving the involved nerve. Post-op recovery was uneventful and devoid of any complications or deficits.

## 3. Discussion

Schwannomas can arise from any nerve which has schwann cells except optic and olfactory nerve as they lack those cells [1].

These tumors are generally encapsulated except from the sinonasal tract and nasopharynx [3].

Most often these tumors are solitary but can occur in multiple areas also. Multiple occurrences are usually associated not only with neurofibromatosis II but also schwannomatosis [4, 5]. Head and neck are the most commonly affected regions (25–45%) with the lateral neck being the frequently involved site [6, 7]. Cytogenetic abnormality of chromosome 22 is seen in 50% cases, and those are associated with neurofibromatosis II [3, 6].

Sinonasal schwannoma contributes about 4% of head and neck schwannomas [8].

It remains asymptomatic for a quite long period because of slow growth. Nasal obstruction is the main symptom of this tumor. Pain, bony dehiscence including skull base and neural symptoms develop later as the tumor enlarges. It can also simulate rhinosinusitis as clinical features are similar [8].

Brachial plexus tumors are rare comprising of only 5% of all tumors of upper limb [9]. The most frequent site is in the head and neck, which comprises 25% of all schwannomas and only about 5% of schwannomas present as brachial plexus tumors [10, 11].

The two most common brachial plexus region tumors are the schwannomas and neurofibromas, both of which are benign and arise from the nerve sheath [12].

Most often, early in the course, minimal neurological deficit will be present, or there will be none at all. Manipulation of the mass can produce paraesthesias or “shocks” in the distribution of the affected nerve, and this can be an important aid to diagnosis. Side-to-side mobility greater than longitudinal mobility of the mass from the nerve is more common in cases of benign lesions, whereas malignancy is associated with firmness and immobility [12, 13]. In cases of brachial plexus lesions, MR imaging is the study of choice to delineate the margins of the tumor from surrounding tissues with greatest contrast [13]. Importantly, however, MR imaging is currently unable to differentiate between schwannoma and neurofibroma [13].

Computerized tomography scanning is optimal at revealing osseous erosion around the spine or changes in neural foramina. A newer modality, MR neurography (MR-magnetic resonance), has the potential to demonstrate the entire course of visualized peripheral nerves [13]. Schwannomas of the vagus nerve grow between the common carotid artery and the internal jugular vein. This growth pattern often leads to a divergence of both structures, which is visible in CT scan or MRI [14].

Cervical vagal schwannomas constitute about 2–5% of neurogenic tumors [3]. These tumors usually present as asymptomatic masses as in our case, if symptomatic patients will present with hoarseness as the main symptom. The sign usually seen in these cases is unilateral vocal cord paralysis. The reported incidence of preoperative vocal cord paralysis is about 12%. Clinical sign elicited in case of vagal schwannoma is a paradoxical cough on palpating the mass due to vagal stimulation. The presence of this sign together with palpation of mass medial to the sternocleidomastoid should arouse the suspicion of a neurogenic tumor [3, 6].

**Figure 11:**
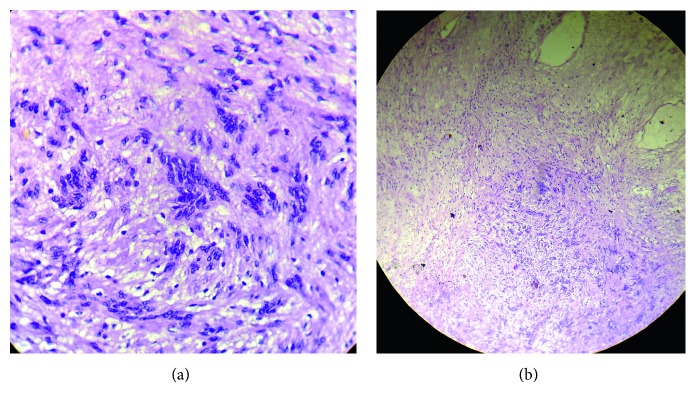
(a) Schwannoma under high power resolution (H&E stain 40x). (b) Schwannoma under low power resolution (H&E stain 10x).

Microscopically (Figure 11), spindle-shaped cells in Antoni-A and Antoni-B arrangement interspersed with Verocay bodies are the characteristic features [2]. These tumor cells typically show a diffuse positive immunoreactivity for S-100 protein [2] and are negative for neurofilament protein. S-100 protein demonstrates a neuroectodermal origin of the tumor [15].

Neurilemmomas are resistant to radiotherapy [2], and as they are well encapsulated, the treatment of choice is conservative surgical enucleation with periodic follow-up. The choice of operation is mainly determined by the relationship between the tumor and the nerve of origin.

**Figure 12:**
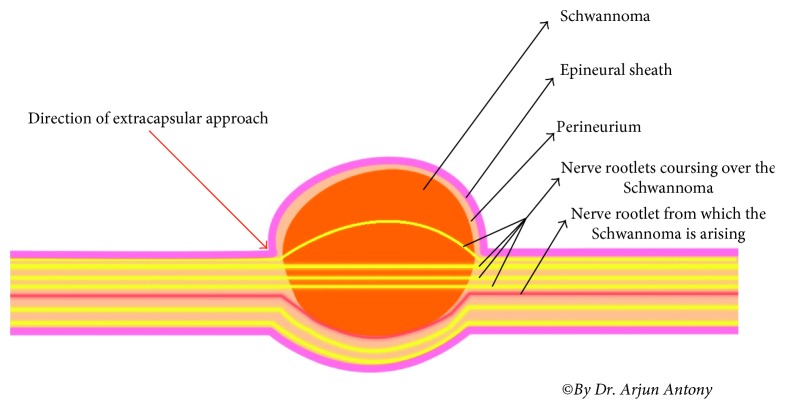
Technique of extracapsular excision.

Conventional extracapsular excision can damage the normal fascicles during dissection of the capsule. Intracapsular excision with gentle dissection between the tumor capsule and normal fascicles minimizes the risk of nerve damage. The epineurial layer covering the tumor capsule should be dissected in a manner similar to peeling an onion to allow for safe removal of the tumor, which should be approached by its proximal and distal poles (Figure 12). The fascicles located in the tumor are usually nonfunctional; therefore, excision of these fascicles does not lead to neurologic deficits [9]. The use of operating microscope and microneurosurgical technique has modified the outcome of conventional extracapsular excision as was seen in our case series, with minimal postoperative morbidity. This technique facilitates extracapsular tumor excision by microscopic technique along with the nerve fibre coursing through it (if it cannot be separated), thus sparing the other nerve fibres, and maintaining minimum morbidity.

Complete extracapsular excision preserving the nerve of origin should be attempted when feasible [16], but for extensive schwannomas, nerve sacrifice with reconstruction and rehabilitation are important considerations.

The choice of surgical approach is dictated by the size of the tumor, its location, its relationship to the great vessels, and the suspicion of malignancy.

## 4. Conclusion

This case report emphasises the rarity of schwannomas in maxillary sinus involving the trigeminal, arising from the brachial plexus, vagus nerve, and cervical spinal nerves, respectively. Among surgical techniques, complete extracapsular excision using microneurosurgical technique with operating microscope is the preferred technique advocated. A schwannoma though rare should be considered as a differential diagnosis of a unilateral slow growing mass in the head and neck region particularly in an adult. They are difficult to diagnose preoperatively. Radiologic findings are usually nondiagnostic. Diagnosis relies on clinical suspicion, and confirmation is often obtained by means of surgical pathology. Thorough preoperative counselling of patients to inform them of the potential occurrence of neurological deficit is important. Long-term surveillance is not recommended, even though these tumors are benign and mostly asymptomatic. Recurrence is rare.
